# The Actionable Innovation Day Approach: Participatory Model for Advancing Critical Care Innovation

**DOI:** 10.2196/73614

**Published:** 2026-05-08

**Authors:** Brett N Hryciw, Cecilia Tran, Rashi Ramchandani, Cameron Love, Christine Caron, Aimee Sarti, Annelise Miller, Suzanne Madore, Michael Chasse, Andy Pan, Simon Didcote, Scott Millington, Heather Galley, Kwadwo Kyeremanteng, Andrew Seely

**Affiliations:** 1 Department of Medicine, University of Ottawa, Ottawa, ON, Canada; 2 Institute of Health Policy, Evaluation and Management, University of Toronto, Toronto, ON, Canada; 3 Department of Family Medicine, University of Ottawa, Ottawa, ON, Canada; 4 Department of Intensive Care, Université de Montréal, Montreal, Canada; 5 Department of Emergency Medicine, The Ottawa Hospital, Ottawa, ON, Canada; 6 Division of Critical Care Medicine, Montfort Hospital, Ottawa, ON, Canada; 7 Ornge (Transport Medicine), Mississauga, ON, Canada; 8 OBS Medical (United Kingdom), Gloucestershire, United Kingdom; 9 Department of Critical Care, Ottawa Hospital, Ottawa, ON, Canada; 10 Clinical Epidemiology Program, Ottawa Hospital Research Institute, 451 Smyth Road, Ottawa, ON, K1H 8M5, Canada, 1 (613) 562-5800; 11 Division of Palliative Medicine, Ottawa Hospital, Ottawa, Canada; 12 Department of Thoracic Surgery, University of Ottawa, Ottawa, ON, Canada; 13 School of Epidemiology & Public Health, University of Ottawa, Ottawa, ON, Canada

**Keywords:** innovation, critical care, interdisciplinary collaboration, patient-centered care, artificial intelligence, AI

## Abstract

Health care innovation is essential for improving patient outcomes, enhancing system efficiency, and preparing for future challenges; however, meaningful progress is often hindered by entrenched barriers such as resistance to change, fragmented interdisciplinary collaboration, and constrained financial and human resources. These persistent obstacles make it difficult for health care systems to translate creative ideas into sustainable, real-world improvements, underscoring the need for structured approaches that support collaboration and reduce implementation friction. To address these challenges, we developed the Actionable Innovation Day (AID) approach, a structured, participatory model designed to generate consensus-based, low-cost recommendations that are feasible for system improvement. The first regional AID event in Eastern Ontario gathered 57 multidisciplinary participants, including clinicians, administrators, patient partners, and industry leaders, for a full-day series of presentations, facilitated discussions, and targeted breakout sessions focused on critical care. Through guided deliberation and collaborative analysis, participants synthesized diverse perspectives into a prioritized set of improvement opportunities. The process yielded 28 actionable recommendations across 4 domains: health care innovation, regionalized care, critical care practices, and the use of artificial intelligence. A postevent survey (86% response rate) showed strong agreement, with 23 recommendations rated above 4 on a 5-point scale. The highest-ranked proposals emphasized the value of strengthening research-industry-clinical partnerships, integrating families more intentionally into intensive care unit rehabilitation and recovery processes, and implementing centralized regional coordination to optimize critical care capacity. Together, these findings illustrate not only the feasibility of the AID model but also the AID model’s ability to surface strategic, context-appropriate solutions that resonate across stakeholder groups. The AID process offers a scalable and adaptable template for advancing health care innovation through collaborative, real-world problem-solving. While this initial event focused on critical care, the underlying principles of structured engagement, iterative consensus building, and interdisciplinary co-design are broadly applicable to many sectors of health care. We encourage institutions, regional networks, and health system leaders to adopt and tailor the AID framework to their own local priorities, recognizing that inclusive innovation processes can accelerate system improvement even in resource-limited settings. Ultimately, the AID approach serves as both a methodology and a call to action: by empowering teams to collectively identify, refine, and champion actionable ideas, health care organizations can build the capacity and culture necessary to drive meaningful and sustained innovation across diverse clinical and operational domains.

## The Current Landscape of Health Care Innovation

Innovation is foundational to the advancement of any discipline, particularly health care. It encompasses technological advancements, process improvements, and cultural and organizational shifts, all of which enhance the quality, efficiency, and accessibility of health care services [1]. As health care systems face challenges related to resource limitations and increased demand, leading to burnout among health care providers, innovative thinking becomes essential to ensure sustainability and progress [2,3].

In health care, innovation acts as a catalyst for improving patient outcomes, optimizing workflows, and enhancing patient experiences [4]. The acute care system in Canada has acknowledged the need to address overcrowded emergency departments, prolonged wait times, and health care provider burnout [3,5,6]. These issues underscore the need for practical innovation to improve both patient care and operational efficiency. However, health care innovation often faces significant barriers, including organizational resistance to change, the complexities of evaluating multiple stakeholder opinions for idea validation, and resource limitations for implementation [7].

## The Actionable Innovation Day Approach as a Catalyst for Innovation

While innovation workshops and ideation events are increasingly used in health care to identify areas for health care innovation, they often lack structured follow-up, fail to produce actionable outputs, or exclude critical stakeholder groups. The Actionable Innovation Day (AID) approach was designed to address these gaps by combining multidisciplinary participation, a mandate for concrete recommendations, and consensus-based validation, all within a scalable, low-cost framework. The AID framework provides a structured model to bring together diverse health care stakeholders to propose actionable changes within a local health authority. The AID approach was conceptually adapted from the Agile Innovation Framework (AIF), which emphasizes iterative, stakeholder-driven development cycles. By embedding this philosophy into a single-day participatory format, the AID approach aimed to translate these principles into a practical model for health care innovation.

The regional AID event aimed to establish a shared understanding of health care priorities and develop practical solutions that can be scaled across regions to drive health care transformation. This paper describes the AID approach through its first implementation, which focused on acute and critical care within a regional health system. Critical care was chosen as the focus due to ongoing challenges related to coordination, resource constraints, and patient outcomes, making it a compelling context in which to evaluate the AID model in practice.

The first regional AID event was implemented with a focus on acute and critical care, engaging stakeholders across the Champlain Local Health Integration Network to address challenges specific to intensive care unit (ICU) and emergency care delivery. Stakeholders invited included physicians, nurses, respiratory therapists, and administrators from hospitals, as well as patient partners, government representatives, and industry participants, thereby allowing for the representation of broad multidisciplinary perspectives. The event was held both in person and virtually to mitigate geographical barriers to participation. It focused on coordinating expedient patient transfers when necessary to provide timely access to appropriate levels of care while minimizing unwarranted risks and expenses of unnecessary patient transfers. Table 1 describes the agenda for the regional AID framework.

**Table 1.** The regional Actionable Innovation Day event agenda.

Time	Session
7 AM-8:15 AM	Coffee and registration
8:15 AM-8:30 AM	Welcome address
8:30 AM-10:00 AM	Speaker presentations: Future Innovation
10 AM-10:30 AM	Networking
10:30 AM-noon	Speaker presentations: Present Innovation
Noon-1 PM	Networking and lunch
1 PM-2:15 PM	Small breakout group discussion: Innovation in Critical Care Regionalization
2:15 PM-2:30 PM	Networking and coffee break
2:30 PM-3:45 PM	Summary of small group discussions
3:45 PM-4:45 PM	Summary and rating of recommendations
4:45 PM-5:00 PM	Closing remarks

The planning committee, composed of representatives from the Ottawa Hospital, the Ottawa Hospital Research Institute, and affiliated academic and industry partners, designed the AID event to foster multidisciplinary collaboration around regional critical care innovation. Speakers were identified through a consultative process that sought representation from key stakeholder groups, including physicians, nurses, respiratory therapists, allied health professionals, hospital administrators, patient partners, and industry leaders. Each invited speaker was asked to deliver a concise presentation, highlighting a current challenge within their domain and concluding with 1 to 3 actionable recommendations for system improvement. The day was structured into two 90-minute presentation blocks, each followed by a brief discussion period to clarify ideas and prepare for the afternoon breakout sessions focused on translating these recommendations into practical solutions. All recommendations were systematically documented by designated recorders (CT and RR) during the AID. Following the morning presentations, participants joined one of three 90-minute small group breakout sessions in the afternoon to explore recommendations to address the key theme of “How to optimize regional critical care delivery?” Participants were randomly assigned to breakout groups, each consisting of 15 people. Moderators facilitated these sessions, helping attendees identify actionable recommendations and items related to the key theme. Recommendations from the breakout sessions were then presented by a small group speaker for a further whole-group discussion and organized into consensus summary recommendations.

At the end of the day, all recommendations were summarized. A follow-up survey using Mentimeter for immediate audience polling allowed attendees to anonymously rate their level of agreement with the suggested recommendations using a 5-point Likert scale. Items with high agreement were prioritized for future implementation.

## The AID Approach in Action: Attendance and Impact

### Participant Demographics

In total, 110 stakeholders were identified and invited to attend the AID event. The attendance rate was 52%, as 57 participants, including invited speakers, attended the regional AID event in person. An additional 10 participants attended the event virtually through Microsoft Teams. Of the 67 people with whom the survey was shared, responses were received from 49 (73%) individuals. Respondents ranged in age from 23 to 65 years, with a median age of 47 (IQR 37‐55) years. The participants included 6 (12.2%) physicians, 2 (4.1%) registered nurses, 1 (2.0%) respiratory therapist, 17 (34.7%) industry partners, 4 (8.2%) allied health care providers, 7 (14.3%) research staff, 5 (10.2%) administrative staff, 5 (10.2%) leadership staff, and 2 (4.1%) patient partners. Gender distribution was 42.9% (n=21) male and 57.1% (n=28) female, and 81.6% (n=40) identified as White. Experience levels ranged from individuals in training to those with over 25 years of experience (Table 2).

**Table 2.** Summary of demographic characteristics of regional Actionable Innovation Day survey respondents (n=49).

Demographic variables	Survey respondents, n (%)
Gender	
Male	21 (42.9)
Female	28 (57.1)
Age group (y)	
<30	4 (8.2)
30‐39	11 (22.4)
40‐49	15 (30.6)
50‐59	13 (26.5)
>60	4 (8.2)
No response	2 (4.1)
Years of experience	
In training	2 (4.1)
0‐5	4 (8.2)
5‐10	4 (8.2)
11‐15	6 (12.2)
16‐20	9 (18.4)
21‐25	13 (26.5)
>25	11 (22.4)
Ethnicity	
White	40 (81.6)
South Asian	3 (6.1)
East or Southeast Asian	4 (8.2)
Black	1 (2.0)
Other	1 (2.0)
Occupations	
Physician	6 (12.2)
Registered nurse	2 (4.1)
Respiratory therapist	1 (2.0)
Allied health care	4 (8.2)
Industry partners	17 (34.7)
Research staff	7 (14.3)
Administration staff	5 (10.2)
Leadership staff	5 (10.2)
Patient partners	2 (4.1)

### Applying the AID Approach Into Critical Care Innovation

#### Overview

The event featured 16 presentations from various professional backgrounds, which covered future innovations in health care, patient-centered care, critical care advancements, nursing and respiratory therapy improvements, and innovations in urban and resource-limited settings. Topics also included the integration of AI, predictive monitoring, effective interhospital transfers, social work innovations, and enhanced partnerships between academia, industry, and health care. Presenters focused on using existing resources to enact meaningful change within the Champlain Local Health Integration Network, and their recommendations were recorded (Table 3).

**Table 3.** Summary of key points and actionable recommendations from presentations of health care professionals of innovation in health care.

Speaker	Title	Recommendation summary
Cameron Love (Hospital CEO)	Future of innovation at TOH^a^	Communicate and instill a sense of pride and importance of discovery within your teams. Create strong program structure and process that incorporates discovery into everything we do and talk about. Identify 3 innovation priorities annually to move forward with.
Kwadwo Kyeremanteng (Chair, Department of Critical Care)	Future of innovation in critical care	Pilot program for wearable monitors in hospitals. Pilot of adopting AI^b^-based early warning system.
Christine Caron (patient representative)	Patient-centered innovation	Alleviate delirium and PTSD^c^ in critical care patients through: ICU^d^ diary (communicates discharge management with diagrams about steps or risks). sounds (headphone and sunlamp): headphone Halo from patients to be protected from sounds in ICU (spa music for physiotherapy, brown noise, and pink noise). physical therapy: teach families OT^e^ or PT^f^ exercises to perform during their ICU stay to mitigate physical impairments or disabilities (eg, ICU cycle and yoga bands).
Annelise Miller (ER MD)	Innovation in resource-limited community or rural ED^g^	Understand the context (who/what/where/why/how) with your community partners. Clearly define providers, materials, time, geographic distance, and physical space. Foster innovation (support local teams or local management, ensure it facilitates timely action, and builds interinstitutional relationship). Innovation should occur alongside advocacy.
Suzanne Madore (Hospital COO and VP)	Innovation in TOH nursing	Foster a culture of innovation with support from leadership. Embed innovation into strategy and comprehend the essence of innovation. Recognize and reward innovation.
Lauralynn McIntyre (ICU Department Research Lead)	Research innovation	Monitor and aim to increase the number of patients enrolled in critical care studies at TOH. Promotion of critical care research at TOH. Training to develop future innovation researchers. Early-career investigator support for innovation researchers.
Matt Bromwich (surgeon, CEO, Hospital Innovation Lead)	Innovation in scientific academic industry partnerships	Innovation should be subtractive (one solution replaces 9 other tasks). Have a team around you (to speak the language and fill in the gaps).
Pierre Cardinal (intensivist, educator)	Innovation in documentation and education	After innovation work and findings on free forums such as Google Firebase Community.
Frank Fiorenza (respiratory therapist, CEO)	Innovation in respiratory therapy	Review mechanical ventilation policy and add a minimum of 1 new solution annually. Identify if ventilation protocols are lung protective Review critical patient transport protocols and add a minimum of 1 solution annually. Do you have an idea for a new device or way to improve patient care—bring it forward. Have you been presented with an innovation recently? Is it viable at your facility? Take on a minimum of 1 new project annually—be the champion.
Michael Chasse (intensivist scientist)	Where is AI in critical care	AI tools should learn to use data “as is”: Tools should use unstandardized data inputs to improve their adaptability and effectiveness in real-world settings. Health care practitioners should prepare for the inevitable partial replacement by AI in specific tasks, embracing the shift as part of the ongoing evolution of medical practice. Anticipate and decide on nonreplaceable tasks: identify and prioritize the tasks that humans should retain, even if AI could perform them more efficiently and/or better.
Andrew Seely (surgeon, intensivist, scientist, CEO)	Monitoring-based predictive decision support	Implement extubation advisor at TOH in a multidisciplinary quality improvement project focused on reducing extubation failure. Continue to seek to get more value from monitoring, by the implementation and evaluation of predictive clinical decision support tools.
Scott Millington (intensivist, VCC^h^ Lead)	Virtual critical care	Hub-and-spoke model for other specialties that deals with any acute patients. Use VCC workflow and technology to take responsibility for region.
Heather Galley (Critical Care SW^i^)	Innovation in social work	Admission checklist → automatic social work referral. Review Critical Care Family Assistance Program criteria and implement updates.
Alexandre Tran (surgeon, intensivist, scientist)	Predicting and preventing ward deterioration	Improve data capture and analysis to personalize the prediction of critical events. Capitalize on the human element to maximize technology effectiveness.
Andy Pan (intensivist, regional transport lead)	Improving interhospital critical care transfers	Involve the transport physician early on and determine the best means of transfer. Pretransfer optimization and minimize transition points.
Andrew Kramer (data scientist) and Simon Didcote (developer, CEO)	Continuous predictive monitoring	Implement continuous noninvasive monitoring outside ICU combined with suitable regulated analytics such as VSI^j^—especially for high-risk patients. Consider investigating use of regulated EWS^k^ such as ViSIG in ICU to improve outcomes. Use a device-agnostic platform to tie analytics together to avoid vendor lock-in.

a TOH: The Ottawa Hospital.

b AI: artificial intelligence.

c PTSD: posttraumatic stress disorder.

d ICU: intensive care unit.

e OT: occupational therapy.

f PT: physical therapy.

g ED: emergency department.

h VCC: virtual critical care.

i SW: social work.

j VSI: Visensia Safety Index.

k EWS: early warning system.

**Figure 1.**
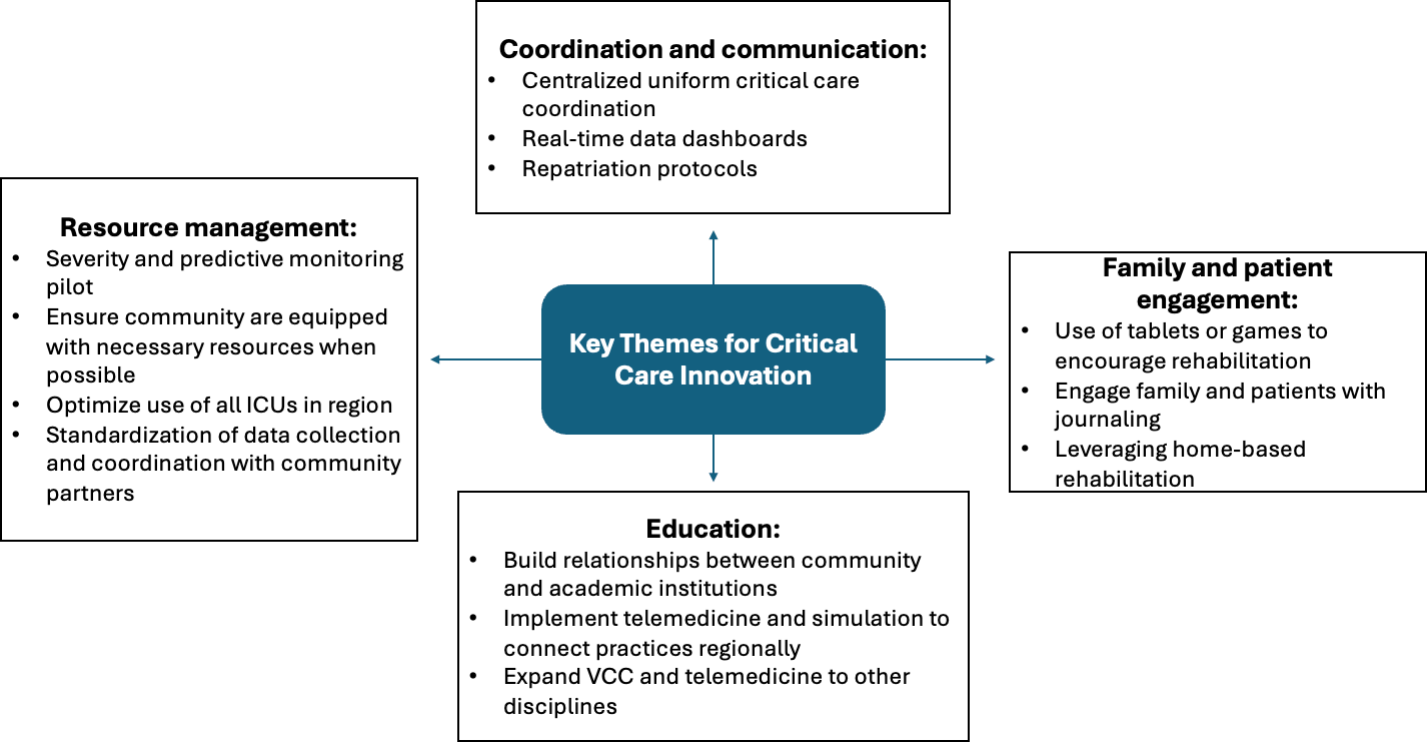
Summary of actionable recommendations to optimize regionalization of critical care for a local health integration network in Ontario, Canada, generated from small group discussions organized by key themes. ICU: intensive care unit; VCC: virtual critical care.

Small group discussions then provided a multidisciplinary platform for further exploring these as well as additional solutions to improve the efficiency and effectiveness of regionalized acute care. In total, 4 themes emerged: (1) coordination and communication, (2) resource management, (3) education, and (4) family and patient engagement (Figure 1). Coordination and communication focused on establishing centralized critical care coordination, real-time data dashboards, and repatriation protocols for optimizing patient transfers. Resource management emphasized ICU utilization, severity, predictive monitoring pilots, and ensuring adequate community resources. Education was highlighted as vital for fostering collaboration among stakeholders, including community members, academic institutions, and health care providers. Family and patient engagement included strategies such as promoting journaling and home-based rehabilitation to enhance patient recovery. All recommendations from the presentations and small group sessions were then synthesized into a comprehensive list of recommendations generated from the event.

#### Actionable Recommendations

A total of 16 speakers provided a total of 42 actionable recommendations during individual presentations on the morning of the AID event, followed by discussion and questions after each. In addition, a set of afternoon parallel small group sessions came up with recommendations to address improved regional coordination of critical care. Subsequently, a total of 28 actionable recommendations emerged from a synthesis of the individual presenters and discussion by participants during the day (Multimedia Appendix 1), as prepared by 2 independent observers (CT and RR) present and recording all events of the day. They summarized the recommendations and discussion into four key domains: (1) innovation in health care, (2) innovation in regionalized care, (3) critical care practices, and (4) use of AI in health care. No preexisting framework was used for classification. A follow-up email survey sent to all participants showed broad support for these recommendations, with agreement ratings ranging from 3.66 to 4.50 on a 5-point Likert scale (Multimedia Appendix 1). Of the 28 recommendations, 23 were ranked greater than 4 and 13 were ranked greater than 4.25. These highly ranked recommendations are included in Table 4.

**Table 4.** Summary of highly ranked actionable recommendations for critical care innovation.

Key domains and recommendation^a^	Rating^b^
Innovation in health care	
Innovation must be fostered through interinstitutional relationships as well as partnerships between research, industry, and health care	4.50
Innovation in regionalized care	
We need to train and mentor early-career investigators to develop future innovation researchers	4.31
We need to promote critical care research in the region	4.26
We need to review policies, protocols, and medical directives to identify 1 to 3 innovation priorities annually to move forward with	4.25
Critical care practices	
Involve families in patient physical therapy in ICU^c^ and in home rehabilitation	4.46
Implement centralized regional critical care coordination to optimize specialized care and bed occupancy	4.44
Implement lung-safe ventilation and follow evidence-based guideline recommendations	4.43
Implement a pilot program for wearable monitors in the region	4.42
Protect patients from sounds in the ICU with the use of headphones or Halo	4.41
Use of AI^d^ in health care	
To avoid vendor lock-in, device-agnostic platforms must be used to tie analytics together	4.32
We must continue to seek to get more value from monitoring, by implementation and evaluation of predictive clinical decision support tools	4.30
We must improve data capture and availability to personalize critical event prediction	4.30
We must identify and prioritize the tasks that humans should retain; we need to capitalize on the human element to maximize effectiveness	4.27

a Recommendations with an overall Likert rating of ≥4.25 were included in the table.

b Rating was evaluated on a 5-point Likert scale.

c ICU: intensive care unit.

d AI: artificial intelligence.

In the domain of “innovation in health care,” participants emphasized fostering partnerships between research, industry, and health care institutions as a key driver of innovation (rating: 4.50). Embedding innovation into organizational strategies received strong support, with a focus on recognizing local innovation efforts and sharing practical tools with related areas to enhance broader applicability. For “innovation in regionalized care,” mentoring early-career investigators and promoting regional critical care research were prioritized, with participants stressing the importance of developing future researchers (ratings: 4.31 and 4.26, respectively). Another key recommendation was to review policies, protocols, and medical directives annually to identify and move forward with 1 to 3 innovation priorities (rating: 4.25). In “critical care practices,” involving families in patient physical therapy and home rehabilitation was highlighted as an important strategy for enhancing patient outcomes (rating: 4.46). Establishing centralized regional critical care coordination to optimize care and bed occupancy also received strong support (rating: 4.44). Additional recommendations included implementing lung-safe ventilation protocols, initiating a pilot program for wearable monitors, and reducing noise exposure in the ICU by using headphones (ratings: 4.43, 4.42, and 4.41, respectively). In the “use of AI in health care” domain, participants highlighted the importance of using device-agnostic platforms to prevent vendor lock-in and promote cohesive data analytics (rating: 4.32). Participants also supported improving data capture and using predictive clinical decision support tools to extract more value from monitoring (rating: 4.30 and 4.30, respectively). Emphasizing the human element by identifying and retaining tasks best suited to humans rather than AI was also considered critical (rating: 4.27).

#### Redefining Innovation in Health Care

Health care innovation is frequently reduced to a radical process that yields disruptive, revolutionary outcomes. In contrast, incremental innovation can represent disciplined, user-centered improvements that address practical challenges [8]. The optimal approach to a sustainable, innovative culture may therefore be achieved incrementally through regular, collaborative assessment of real problems faced by health care providers, patients, and institutions [9]. The regional AID event embodies this definition of innovation, focusing on low-cost, actionable improvements designed to enhance patient outcomes and health care system efficiency. Unlike typical innovation workshops that emphasize idea generation alone, the AID event formalizes the process by requiring structured output, involving multilevel stakeholders, and using postevent consensus polling. These features position the AID framework as a replicable model for driving practical innovation across health care settings, although the inaugural event focused on critical care delivery.

Furthermore, the AID framework’s participatory model promotes easily implemented ideas that are subsequently positioned for rapid iteration and validation based on real-world feedback. By centering on the immediate needs of stakeholders, the AID event moves away from the often imagined “lightbulb moment” of innovation toward a collaborative, methodical approach that balances creativity with practical implementation. Nonetheless, it is recognized that subsequent implementation of AID is a significantly greater barrier.

#### Building on the AIF

The regional AID event shares many parallels with and builds on the principles of Agile Innovation, which emphasizes iterative cycles of planning, execution, and adaptation [10]. Within the AIF, the fifth step includes an innovation forum—a structured design event that brings various stakeholders to brainstorm potential innovations. The AID model represents an enhanced and expanded version of this forum. In the outlined AID approach, stakeholder engagement was the first critical component. By involving individuals from multiple disciplines, including those who control resources and those directly affected by care, the AID approach ensured that the identified problems had practical and systemic significance. Our process begins with presentations from local experts, which provide the necessary context and highlight the nuances of specific challenges within their domains. These expert-driven insights were crucial in framing the problems effectively and in proposing potential solutions tailored to real needs. The stakeholders conclude their presentations with actionable recommendations informed by the literature, professional experience, and/or expert opinion. This structured knowledge-sharing phase frames the problem effectively and seeds the ideation process. Following the presentations, we facilitated group breakout sessions that enabled stakeholders to refine the presented solutions and participate in a creative, judgment-free ideation session. These sessions provided a multidisciplinary platform for the exploration of innovative approaches and the assessment of the feasibility of each idea. The collective prioritization of actionable recommendations allowed the AID contributors to establish a foundation for subsequent development and validation phases of the AIF.

Beyond its direct impact on critical care, the AID approach has broader relevance, given the pressing challenges of an aging population and rising health care expenditures. With the projected increase in chronic diseases and long-term care needs, health care systems require structured, multidisciplinary approaches that can rapidly identify and implement cost-effective, sustainable solutions. The iterative nature of the AID framework allows for practical, system-level interventions that can be adapted to various domains, from geriatrics to community-based preventive health programs.

#### Overcoming Conventional Barriers to Innovation

Systemic barriers often impede health care innovation [11]. The AID approach deliberately addressed these barriers by incorporating specific strategies designed to facilitate a conducive environment to implement change. One significant barrier to health care innovation is the misalignment between administrative priorities and the adaptive, rapid changes required for practical innovation [11]. Policymakers and administrators may be reluctant to embrace such changes due to concerns about potential risks, costs, or disruptions to established processes. The AID event actively involved administrative leaders throughout the event to align innovation priorities with organizational goals, enabling smoother adoption and increasing the likelihood of long-term integration. Furthermore, the intention to promote incremental innovation, as opposed to radical invention, ensures that the implementation process can be more readily navigated. A second substantial barrier involves the limited availability of financial and infrastructure resources [7]. High-cost innovations can be particularly challenging to implement in resource-constrained environments such as Canadian health care. The AID framework addressed this challenge by emphasizing low-cost, high-impact solutions that required minimal resource investment. This approach not only made the proposed innovations more feasible for a wide range of institutions but also reduced barriers related to resource allocation, thereby promoting more equitable innovation. Third, the diffusion of innovation is another critical aspect of sustainable health care change [12,13]. The AID approach promoted the diffusion of recommendations in several ways. The recommendations are naturally distributed throughout the health authority by involving innovators and early adopters across most disciplines and from sites across the region. Similarly, because of the collaborative approach to developing the recommendations, those involved are more likely to appreciate the relevance and alignment of the proposed solutions to the specific context of their local community. Finally, by inviting administrators, we hope to promote a cultural shift that promotes innovation from those in leadership roles. Leaders who actively participate in and advocate for innovative initiatives set a positive example, foster a supportive culture, and help embed innovation as an organizational value [14]. Barriers such as resistance to change often hinder the adoption of new practices. This resistance can be exacerbated by fears that new innovations will add to the workload of already overburdened health care teams. The AID framework sought to counter these concerns by focusing on innovations designed to streamline workflows and reduce unnecessary burdens. It is anticipated that approaches such as the AID framework could help foster a more positive perception of innovation, thereby encouraging broader acceptance and adoption among health care teams.

#### From the AID Approach to Implementation: Next Steps in the AIF

The AIF provides a pathway for advancing the actionable recommendations from the regional AID event beyond early development to institutional or regional integration. The AID approach corresponds to the innovation forum described in the fifth stage of the AIF, serving as an initial platform for generating and refining innovative ideas. However, to move these ideas toward real-world implementation, the subsequent AIF stages, which are focused on continuous iteration, refinement, and early implementation, must be undertaken. These stages facilitate “development sprints” that iterate rapidly on solutions, incorporating ongoing feedback to prepare innovations for broader evaluation and deployment [10]. For these next steps, ongoing communication and collaboration among AID event participants would be a cornerstone of success. This will ensure the development of practical, context-specific solutions that can move forward effectively. The AID model can also be conducted at regular intervals to maintain momentum and evaluate progress. Future iterations of the AID event can facilitate follow-up discussions on implementation progress, allowing for both synchronous and asynchronous advancement of innovations.

## Limitations

One of the challenges during a communal collegial effort to distill consensus recommendations is the need to summarize information during a discussion process into a summary list of recommendations. This is variably facilitated by a chair and therefore lacks standardization. This would be a limitation of this work and the method in general. In addition, a synthesis of recommendations may also be subject to typically unconscious bias. Nonetheless, there are recognized best practice means to amalgamate and synthesize information, which would benefit this type of single-day collaborative educational event designed to generate consensus recommendations. The Delphi method is a validated, iterative consensus-building technique in which experts anonymously rate and refine a series of statements across multiple rounds. When used after an innovation conference, it provides a systematic means to transform raw, diverse input into consolidated, prioritized, and consensus-informed recommendations, reducing bias while ensuring broad stakeholder alignment.

## Moving Forward: The Role of the AID Framework in Health Care Innovation

The AID process represents a possible organizational format for a meeting to engage health care leaders on the topic and process of innovation, supporting the generation of consensus recommendations and hopefully serving as a catalyst for innovation that improves care. The design is aimed to create a participatory model that engages multidisciplinary and multilevel stakeholders. By aligning with the AIF, a structured, practical approach focused on low-cost, scalable solutions to advancing critical care is provided, with the aim of developing innovations that are both impactful and sustainable. However, we appreciate that the next steps are to follow through on the recommendations, that is, turning recommendations into action, which is the admittedly more challenging next step of implementation. The AID process offers a practical framework for initiating, developing, and refining innovation within health care. Although this approach was demonstrated in critical care, we encourage health care leaders and stakeholders to adapt the AID process to their own specialties and local contexts by engaging relevant partners within their institutions and regions. Ultimately, the AID event approach can be flexibly applied and scaled to support innovation across diverse health care settings.

## Supplementary material

10.2196/73614 Multimedia Appendix 1 Attendee agreement ratings for recommendations related to (1) innovation in health care, (2) innovation in regionalized care, (3) critical care practices, and (4) use of artificial intelligence in health care. Agreement was rated on a Likert scale (0=no agreement and 5=strong agreement).

## Abbreviations

AID: Actionable Innovation Day
AIF: Agile Innovation Framework
ICU: intensive care unit
