# Supplementary figures and images for: The Actionable Innovation Day Approach: Participatory Model for Advancing Critical Care Innovation
Source: J Med Internet Res. 2026 May 8;28:e73614. doi: 10.2196/73614 (PMC13155498; doi:10.2196/73614)

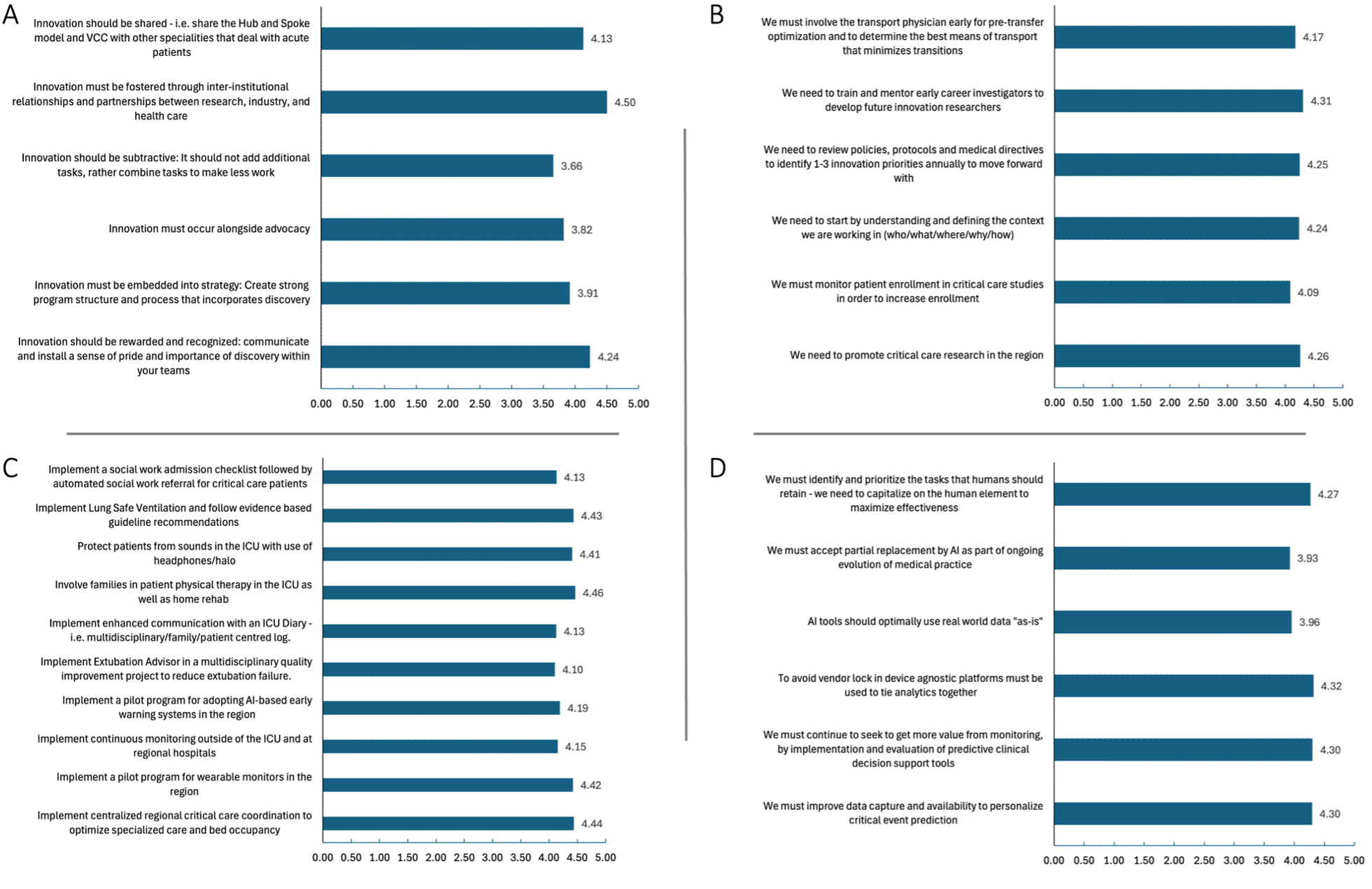

Supplement: Multimedia Appendix 1 [file jmir-v28-e73614-s001.png]
